# Cumulative effect of risk and protective factors on unintentional injury for Chinese rural children: a nested case-control study

**DOI:** 10.1186/s12889-021-11769-7

**Published:** 2021-09-23

**Authors:** Hui Zhang, Fengxin Bai, Hongling Song, Jun Yang, Xinlong Wang, Qingfang Ye, Yuqiu Zhou

**Affiliations:** 1 School of Nursing, Harbin Medical University, No. 39 Xinyang Road, Gaoxin District, Daqing City, 163319 Hei Longjiang Province China; 2 Liming Community Health Service Center of Daqing People’s Hospital, Daqing City, Hei Longjiang Province China; 3 English Department, Harbin Medical University (Daqing), Daqing City, Hei Longjiang Province China

**Keywords:** Unintentional injury, Rural children, Risk factors, Cumulative effect, Nested case-control study

## Abstract

**Background:**

Childhood unintentional injuries are the leading cause of death and disability for children. Despite the risk factors that lead to the occurrence of injuries have been identified, the relationship between cumulative effect of risk/protective factors and unintentional injuries is unclear. The aim of this study was to explore the cumulative effect of risk factors as well as protective factors and their interaction on unintentional injury to rural children.

**Methods:**

We used a nested case-control study design from a cohort database. The study comprised 1696 children aged 6 to 14 years. Among them, 424 were cases with unintentional injury and 1272 were their matched control. After controlling for the significant sociodemographic variables, linear and logistic regression analyses were performed.

**Results:**

The risk of unintentional injury increased with the increasing number of risk factors - RFI from 1 to 3 (OR_RFI(1)_ = 0.978, 95% CI 0.739–1.296), (OR_RFI(2)_ = 1.720, 95% CI 1.233–2.397), (OR_RFI(3)_ = 5.162, 95% CI 3.129–8.517). PFI (1) was associated with lower risk of injury, but this association was at the edge of significance (*p* = 0.052). The increased risk in those with PFI (2) was not significant (*p* = 0.254). The severity of the unintentional injury significantly increased with the increasing number of the risk factors (*p* < 0.01), and significantly decreased with both the increasing number of protective factors (*p* = 0.001) and interaction of the risk and protective factors (*p* < 0.01).The interaction of RFI and PFI could explain 32.2% of the unintentional injury severity.

**Conclusions:**

According to the findings of the present study, cumulative risk factors and protective factors, as well as their interaction were associated with the occurrence and/ or severity of unintentional injury in children.

**Supplementary Information:**

The online version contains supplementary material available at 10.1186/s12889-021-11769-7.

## Background

Childhood unintentional injuries are the leading cause of death and disability for children aged 0 to 14 in the world [1]. In 2017 the Global Burden of Diseases project estimates over 2 million children died from injury in the whole world, equivalent to 5581 child deaths per day and almost four per minute [2]. Over 95% of global child injury deaths occur in low- and middle-income countries [3]. In China, thousands of children die from unintentional injury each year; the incidence rate of injury is from 10.93 to 64.3%, and it is the leading cause of mortality for children between the ages 1 and 14 [4, 5]. In the United States, unintentional injury killed over 11,000 children in 2017, over 6.7 million children were treated in the emergency department, the financial toll of child injuries exceeded $96 billion annually [6]. Unintentional injuries threaten children’s health and life, and bring heavy economic and psychological burden on families and the whole society. Fang et al. reported that the overall economic burden of injury incident in 303 students of elementary and secondary school over 1 year, in Xiamen, China, was 1,014,649 RMB (148,6667 USD) total, 3348 RMB (491 USD) per capita, and 2779 RMB (407 USD) per incident [7]. Thus, childhood unintentional injuries have become a prominent and focus issue in the field of public health.

In China, childhood unintentional injuries are more prominent in rural areas. The unintentional injury risk of death in rural areas was approximately 1.95 times that compare to urban areas of China [8, 9]. In Chinese rural areas, there is a special group called left-behind children whose parents have left the hometown for work in the urban while their children stay with their grandparents in rural hometown. There are 104 million children living in rural area, and 60 million of them are left behind children [10]. Studies revealed that left-behind children had higher incidence of unintentional injury in China because of lacking of security guidance and protection from their parents [11]. Many researches indicated that the risk of unintentional injury in children is affected by children’s personal attributes (age, gender, harmful behavior, behavior problems), family environment (the number of siblings, economic status and environmental facility, education of primary caregiver and supervision, the knowledge and skills of primary caregivers of preventing injury), as well as by other factors of children’s social environment [12, 13]. Boys, with schizoid behavior problem, anxiety/depression and hyperactive, risk-taking behaviors are more likely to suffer from unintentional injury [13, 14]. Primary caregivers with low supervision, lacking knowledge or skills for preventing injuries could increase the children’s risk of injury [12, 15]. Previous studies had only focused on risk factors of unintentional injuries in childhood, but few had concerned the relationship between the cumulative effect of risk/protective factors on unintentional injuries. The occurrence of an injury event is the result of multiple factors, not just risk factors. Rutter proposed the cumulative effect to access these multiple factors which may helpful to explain interaction effect [16]. The cumulative effect of risk/protective factors is widely used in developmental psychology to consider the power of multiple factors exposure effects. Some researchers adopted the Risk Factor Index (RFI) and Protective Factor Index (PFI) to measure the cumulative effect of risk and protective factors, and their interactive effect on the health [17, 18]. Hence, the current study aimed to determine the relationship of the cumulative effect of risk and protective factors and their interaction with unintentional injury in rural children aged 6 to 14. We hope that our study will contribute to the understanding of the cumulative effect of risk/protective factors in childhood unintentional injury which provides a new point to design the most effective injury prevention and intervention for childhood.

## Methods

### Study design, setting and participants

The present study is a nested case-control study performed from within the prospective study “PCUI-Protection for Childhood unintentional injury in rural areas in Hei Longjiang Province China”. The named cohort study started in 2017 and comprised 3 rural regions from the Hei Longjiang Province chosen according to the economic development level: Daqing region, Qiqihar region and Jia Musi region. Random cluster sampling was used to select 12 elementary and junior schools from these regions and the total sample comprised 3163 children. Data were collected on some sociodemographic and other characteristics of children, their families and caregivers, data about risk and protective factors for unintentional injuries. All data were obtained from primary caregivers.

All study subjects were followed up for 12 months and a record of unintentional injury was collected. Children aged 6 to 14 were selected for the study. Children who had a record of unintentional injury in the last 12 months were recruited in the case-group, and the controls were similarly aged children who didn’t suffer from unintentional injury over the same period. To ensure homogeneity of subjects, controls were selected from the same region and the same class. For each case three controls were selected from the cohort database to yield matching ratio 1:3. The total sample size for the study was 1696 children (424 cases and 1272 controls).

### Measures

Both sociodemographic (age, gender, father and mother’s ages and education, primary caregiver’s education and health status, left-behind children, household income) and the characteristics of childhood unintentional injury were collected. Unintentional injury was defined as an injury that (a) was diagnosed as a non-fatal injury by physicians and received medical treatment or (b) received emergency medical treatment or assistance from teachers, parents, classmates or others, and (c) required the child to rest for more than half a day [19]. According to the international classification of diseases (ICD-10) the types of injury were classified into fall, traffic injury, burn, cut injury, blunt force and percussion injury, animal bite injury, poisoning, drowning, and others (electrocution, suffocation, fire injury, and frostbite). The severity of the injury was also collected through the numerical assessment (0 = none to 10 = extremely serious) [20]. Risk and protective factors were identified for childhood unintentional injury in the previous study [21]. Risk and protective factors were exposure variables, the severity of unintentional injury was outcome variable, meanwhile sociodemographic were potential confounders in this study.

### Risk factors

#### Strengths and difficulties questionnaire (SDQ)

We used a parent report questionnaire which assesses the children’s behavior problems. This questionnaire consists of 25 items, including five subscales: emotional problems, conduct problems, hyperactivity/attention deficit, peer interaction problems and prosocial behavior [22]. Each item is rated on three-point Likert scale (0 = inconformity, 2 = absolutely conformity). The total score ranges from 0 to 100, with the higher scores indicating the more serious behavioral problems. In this study, the Cronbach’s α was 0.873.

#### Injury behavior checklist (IBC)

This scale is a parent report that assesses the children’s harmful behavior. It contains 24 items, and each item is rated on a five-point Likert scale (0 = never to 4 = always). The total score ranges from 0 to 96, with the higher score indicating the more risky behaviors the children took [23]. The Cronbach’s α was 0.90 in this study.

#### Perceptions of risks and hazards (PRH)

This scale is a parent report that assesses the perception of children’s injury risk. It contains 8 items, each item is rated on a five-point Likert scale (1 = impossible to 5 = extremely possible), the total score ranges from 8 to 40; with the higher score indicating the greater the likelihood of injury [24]. The Cronbach’s α was 0.73 in this study.

### Protective factors

#### Parent supervision attributes profile questionnaire (PSAPQ)

The PSAPQ contains 29 items covering four subscales: protectiveness, supervision beliefs, tolerance for children’s risk-taking, and belief in fate. Each item is a five-point Likert scale (1 = never to 5 = all of the time). The total score ranges from 29 to 145. The higher the score, the more supervised the children were by the primary caregiver [25]. The Cronbach α was 0.86 in this study.

#### Home observation for measurement of the environment (HOME)

This scale assesses the safety of home environment. It contains 8 items, each item is rated on 0 (None) and 1 (Yes). The total score ranges from 0 to 8. The higher the score, the more safe of home environment the children had [26]. The Cronbach α was 0.85 in this study.

#### Knowledge, attitude and skills questionnaire for children unintentional injury (KAP)

This scale was developed specifically for this study through parents interview, focus group guide and expert Delphi. The content validity of this scale was 0.87. It is a parent report that assesses the knowledge, attitude and skills of children unintentional injury (English version seen Additional file 1). It contains 49 items covering three subscales: knowledge, attitude and skills. Knowledge subscale has 18 items, each item is rated on 0 to 2 (0 = Don’t know, 1 = Yes, 2 = None). The subscale of attitude has 12 items, each item is rated on five-point Likert score (1 = extremely disagree to 5 = absolutely agree). The subscale of skills has 19 items, each item is rated on five-point Likert score (1 = never to 5 = always). The total score ranges from 31 to 191. The higher the score, the more knowledge, attitude and skills of parents had [21]. The Cronbach α was 0.85 in this study.

### Risk and protective factor indices

In this study, the scores on each measure were dichotomized based on the cumulative risk model which defined binary risk/protective factors (risk/protection versus no risk/no protection). Each singular risk/protective categorical is accomplished by a statistical criterion (e.g. upper quartile of risk exposure = 1; all others = 0) and then summed together to form the risk/protective factors index [9, 12, 27, 28]. The risk factor index (RFI) was computed by adding on 3 risk measures: SDQ ≥ 22 (1 point), IBC ≥ 17 (1 point), and PRH ≥ 18 (1 point). The protective factor index (PFI) was computed by adding on 3 protective measures: PSAPQ ≥105 (1 point), HOME ≥6 (1 point), and KAP ≥ 151 (1 point). RFI(1) means the presence of only one out of 3 risk factor scale measures, RFI(2) means the presence of any two of them, and RFI(3) means the presence of all three risk factor scale measures. Also the same to the PFI.

### Statistical analysis

SPSS version 25.0 was used for the statistical analysis. Characteristics of cases and controls were described using mean value (standard deviation, SD) or frequencies and percentages. Independent sample’s t-test, Fisher’s one way ANOVA test, Chi-square and spearman correlation analyses were conducted to test univariate and bivariate significance, and *p* value < 0.05 was considered a statistical significance. Each RFI and PFI subtracted mean for centralization/standardizing respectively, and then RFI ⨯ PFI was computed [29]. Logistic regression analysis was used to evaluate the relationship between RFI, PFI and their interaction (RFI x PFI) and unintentional injury. As the severity of unintentional injuries was normally distributed, the linear regression was performed to assess its association with RFI, PFI and their interaction. The significant sociodemographic variables have also been controlled during the logistic and linear regression. Children who had missing record of unintentional injury in the last 12 months were excluded.

## Results

### Characteristics of samples

Three thousand one hundred sixty-three children were enrolled in this cohort database, and all the subjects were completed 12 months followed-up and had a record of unintentional injury. In the present study 424 children with unintentional injury were compared with 1272 children without unintentional injury during the same period of observation. The incidence of unintentional injury was 13.4% in rural children. Out of children with injury, 26.9% (116) had experienced fall, 17.9% (76) had accidental injury, 16% (68) had animal bite injury, 13.7% (58) had burn, 10.4% (44) had cut injury, 8.3% (35) had injury by blunt object and percussion, 3.5% (15) had poisoning and 3.3% (12) had other type of injuries. The mean severity of injury was 2.82 ± 2.35. Some characteristics of cases and controls are presented in Table 1. Since cases and their controls were chosen from the same class of the same school, they were of similar age. There was also no difference in gender or the education level of their mothers. The two study groups significantly differed in the percentage of left behind children (*p* < 0.01), the person who acted as primary caregiver (*p* < 0.01), education (*p* < 0.05) and health (*p* < 0.05) of the primary caregiver, mother’s and father’s age (*p* < 0.05), father’s education (*p* < 0.01) and household income (*p* < 0.01).

**Table 1:** Characteristic of cases with unintentional injury and their controls, children 6 to 14 years old, Hei Longjiang, China, 2017–2018

Characteristics	Case group (***n*** = 424)	Control group (***n*** = 1272)	Statistics
**Age (year) M (SD)**	10.93 (1.76)	11.07 (2.02)	t = 1.353
**Gender (%)**			χ^2^ = 2.216
**Boy**	213 (50.2%)	586 (46.1%)	
**Girl**	211 (49.8%)	686 (53.9%)	
**Left behind children (%)**	220 (51.9%)	453 (35.6%)	χ^2^ = 35.185^**^
**Primary caregiver (%)**			F = 35.255^**^
Mother	157 (37.0%)	908 (71.4%)	
Father	21 (5.0%)	115 (9.0%)	
Grandparents	235 (55.4%)	235 (18.5%)	
Baby sister or others	11 (2.6%)	14 (1.1%)	
**Primary caregiver’s education**			F = 6.008^*^
Illiteracy	27 (6.4%)	39 (3.1%)	
Elementary school	100 (23.6%)	278 (21.9%)	
Junior high school	233 (54.9%)	754 (59.3%)	
Senior high school	57 (13.4%)	152 (11.9%)	
Above college	7 (1.7%)	49 (3.9%)	
**Health of primary caregiver**	8.68 (2.47)	9.14 (3.99)	t = 2.232^*^
**Mother’s age**	36.56 (6.00)	37.51 (6.00)	t = 2.977*
**Mother’s education**			F = 0.050
Illiteracy	21 (5.0%)	56 (4.4%)	
Elementary school	81 (19.1%)	291 (22.9%)	
Junior high school	255 (60.1%)	711 (55.9%)	
Senior high school	53 (12.5%)	164 (12.9%)	
Above college	14 (3.3%)	50 (3.9%)	
**Father’s age**	38.19 (6.07)	39.18 (5.60)	t = 2.944*
**Father’s education**			F = 110.423^**^
Illiteracy	5 (1.2%)	3 (0.2%)	
Elementary school	87 (20.5%)	30 (2.3%)	
Junior high school	275 (64.9%)	953 (74.9%)	
Senior high school	46 (10.8%)	220 (17.3%)	
Above college	11 (2.6%)	66 (5.2%)	
**Household income (per person per month in yuan)**			F = 7.892**
< 1000 RMB	50 (11.8%)	83 (6.5%)	
1000–3000 RMB	241 (56.8%)	442 (34.7%)	
3001–5000 RMB	97 (22.9%)	569 (44.7%)	
> 5000 RMB	33 (7.8%)	178 (14.0%)	

*p < 0.05 ** *p* < 0.001

### Risk factors and protective factors, and their association with unintentional injury

The comparison of risk factors and protective factors between the two groups was presented in Table 2. The total scores of SDQ, IBC and PRH were significantly higher in the case group than in the control group (*p* < 0.01). On the contrary, the total scores of HOME and KAP were significantly lower in the cases than in their controls (p < 0.01). PSAPQ scores did not significantly differ between comparison groups. The numbers and percentages of RFI and PFI in the case group and control group were presented in the Table 3. The number of risk factors in the case group was higher than in the control group. In the case group children had the most frequent RFI (1) - 35,4% or RFI (2) - 20.5%, whereas their controls had the most frequent RFI (0) - 47.8% or RFI (1) - 35.7%. These differences were significant (*p* < 0. 01). The comparison groups did not significantly differ in PFI (*p* > 0.05).

**Table 2:** Risk and preventive factors in cases with unintentional injury and their controls, children 6 to 14 years old, Hei Longjiang, China, 2017–2018

Variables	Case group (***n*** = 424)	Control group (***n*** = 1272)	t
Risk factors			
SDQ	20.58 ± 7.06	18.78 ± 7.78	−4.227**
IBC	16.37 ± 13.94	11.31 ± 11.98	−7.226**
PRH	15.98 ± 7.04	14.08 ± 7.36	−4.646**
Preventive factors			
PSAPQ	91.06 ± 20.50	90.20 ± 22.83	−0.693
HOME	5.99 ± 1.42	6.37 ± 1.11	5.099**
KAP	133.09 ± 21.65	136.84 ± 21.37	3.125**

*SDQ* Strengths and Difficulties Questionnaire, *IBC* Injury Behavior Checklist, *PRH* Perceptions of risks and hazards, *PSAPQ* Parent Supervision Attributes Profile Questionnaire, *HOME* Home Observation for Measurement of the Environment, *KAP* Knowledge, attitude and skills for children unintentional injury. ** *p* < 0.01

**Table 3:** Distribution of RFI and PFI in cases with unintentional injury and their controls, children 6 to 14 years old, Hei Longjiang, China, 2017–2018

Factors	Case group (***n*** = 424) Number (%)	Control group (***n*** = 1272) Number (%)	Statistics
RFI			F = 85.706**
0	119 (18.1%)	608 (47.8%)	
1	150 (35.4%)	454 (35.7%)	
2	87 (20.5%)	175 (13.8%)	
3	68 (16.0%)	35 (2.8%)	
PFI			F = 1.866
0	282 (66.5%)	762 (59.9%)	
1	82 (19.3%)	344 (27.0%)	
2	60 (14.2%)	166 (13.1%)	

*RFI* Risk Factors Index, *PFI* Protective Factor Index; ** *p* < 0.01

According to Spearman correlation analyses, unintentional injury had a positive significant correlation with the RFI (r_s_ = 0.181, *p* < 0.01) and had a negative significant correlation with the PFI (r_s_ = − 0.051, *p* < 0.05) and RFI ⨯ PFI (r_s_ = − 0.113, *p* < 0.01). In Table 4, after controlling for the significant sociodemographic variables (left-behind children, primary caregiver, education and health of primary caregiver, mother’s age, father’s age and education, and household income), the occurrence of unintentional injury significantly increased with the increasing number of risk factors. The risk of unintentional injury increased with the RFI from 1 to 3 (OR_RFI(1)_ = 0.978, 95% CI 0.739–1.296), (OR_RFI(2)_ = 1.720, 95% CI 1.233–2.397), (OR_RFI(3)_ = 5.162, 95% CI 3.129–8.517). The risk of unintentional injury was 3 times when RFI was 3 than 2, the risk occurrence of unintentional injury was 1.76 times when RFI was 2 than 1. In addition, the PRF (1) was associated with lower risk of injury, but this association was at the edge of significance (OR = 0.746, *p* = 0.052). The increased risk in those with PRF (2) was not significant (OR = 1.224, *p* = 0.254).

**Table 4:** Cumulative effect of risk factors and protective factors on unintentional injury^a^ in children 6 to 14 years old, Hei Lingijand, China, 2017–2018

	OR	95%CI		*p*
		Low	High	
RFI				< 0.01
RFI (1)	0.978	0.739	1.296	0.878
RFI (2)	1.720	1.233	2.397	0.001
RFI (3)	5.162	3.129	8.517	< 0.01
PFI				0.038
PFI (1)	0.746	0.555	1.003	0.052
PFI (2)	1.224	0.865	1.733	0.254

^a^According to logistic regression analysis. Odds Ratios were adjusted for significant sociodemographic variables (left-behind children, primary caregiver, education and health of primary caregiver, mother’s age, father’s age and education, and household income); RFI(1): the presence of only one out of 3 risk factor scale measures; RFI(2):the presence of any two of them; RFI(3): the presence of all three risk factor scale measures; PFI(1): the presence of only one out of 3 protective factor scale measures; PFI(2):the presence of any two of protective factor scale measures; RFI(3): the presence of all three protective factor scale measures

### The severity of unintentional injury and RFI, PFI and interactive effect

According to the results of the linear regression analysis, presented in Table 5, the severity of the unintentional injury significantly increased with the increasing number of the risk factors (*p* < 0.01), and significantly decreased with both the increasing number of protective factors (*p* = 0.001) and interaction effect of the risk and the protective factors (*p* < 0.01). The effects of RFI x PFI could explain 32.2% of the unintentional injuries severity.

**Table 5:** Relationship between RFI, PFI and their interaction (RFI x PFI) with the severity of unintentional injury^a^ in 424 children 6 to 14 years old, Hei Longjiang, China, 2017–2018

	Step 1		Step 2		Step 3	
	β	p	β	p	β	p
Constant		0.013		< 0.01		< 0.01
RFI	0.278	< 0.01	0.269	< 0.01	0.256	< 0.01
PFI			−0.053	0.009	−0.071	0.001
PFI ⨯ RFI					−0.074	< 0.01
F	178.277	< 0.01	92.826	< 0.01	66.448	< 0.01
△R^2^	0.072		0.075		0.080	
Adjusted R^2^	0.316		0.318		0.322	

^a^According to linear regression analysis. *RFI* Risk Factors Index, *PFI* Protective Factor Index, *PFI ⨯ RFI* the interaction of RFI and PFI

## Discussion

The present study is the case-control, nested in the large cohort study. It comprised all children who had unintentional injuries during 1 year, the incidence of unintentional injury being 13.4% which is higher than the early reports for rural children aged 5 to 16 (10.93%) and left behind children (11.21%) in China [5, 30]. Perhaps one reason is that in the case group 55.4% primary caregiver are grandparents who have poor health status and they are not suitable for supervising children. They also lacked knowledge and skills on prevention for injury. Once children suffered unintentional injury, they couldn’t respond and take correct first aid on time [30, 31]. Another reason is that different regions have different social and economic development levels. The gross domestic product (GDP) of Hei Longjiang Province is low in China [32]. The environmental facilities are not safe and the transportation of rural areas mainly rely on electric vehicles and agricultural locomotives [33]. The fall and traffic injury are the most common unintentional injury in this study. Hei Longjiang province is located in the northeast of China and its winter season is too long, the rural households need to make fires for heating, drinking water, cooking and other daily activities, so children are prone to burns [34]. Previous studies showed that boys were more likely to experience injury than girls in all age groups [35, 36]; however, there was no significant difference between boys (50.2% %) and girls (49.8%) in the case group in this study. This could be the result of different attitude of parents toward their children, the boys being more often punished for their risk-taking behavior, whereas the same behavior of girls is tolerated [37, 38].

The aim of this research was to explore the single effect and cumulative effect of risk factors (children’s behavior, parents’ risk perception) and protective factors (parental supervision, parents’ first aid knowledge, attitudes and skills, family environment) on the occurrence of unintentional injuries. The more prominent the children’s behavioral problems and harm behaviors and the worst parents’ perception of injury risk, the more severity of childhood injury. Moreover, the children’s behavioral problems and risk-taking behaviors can predict the occurrence of unintentional injury [39]. Children with behavioral problems have a high incidence of unintentional injuries. Children’s behavioral problems have predictive effects on the unintentional injuries, especially those as antisocial, aggression, anxiety/depression, hyperactivity and discipline violation [14]. The cumulative effect of risk factors and protective factors play an important role in the childhood unintentional injury. In the present study, the results of logistic regressions showed that with the increasing number of RFI, the risk of unintentional injury increased. In addition, the risk for the occurrence of injury was lower in those with PFI (1) in comparison to those without any of protective factors. Also, the risk and protective factors were related to the severity of unintentional injury. The linear regression analysis indicated that risk factors and protective factors had interactive effect to each other, protective factors could regulate the effect of risk factors, and with the increased number of protective factors, the effect of risk factors will be weakened. The cumulative effect on co-occurring and multiple risk or protective factors have been concluded in children and adolescents with behavioral problems which indicate the more risk factors they are exposed to, the worse the outcome is [16, 40, 41].

The current study is the first to describe the relationship between exposure to cumulative effects of multiple risk/protective factors and unintentional injury in rural children through the nested case-control study. It contributes to a new view point to the risk factors and prevention strategies for the occurrence of rural childhood unintentional injury in China. Specifically, it reveals significant cumulative effect of risk and protective factors in the rural childhood injury. Besides the known disadvantages of the used type of the study, the present investigation has several limitations. First, only a few risk factors and protective factors were investigated, and some more important factors may be missed. Second, the data were collected from primary caregivers, so there is possibility that some social bias and recall bias were present. Third, the severity of injury was recorded by the primary caregiver, from 0 to 10 numerical assessment, which may influence the accuracy of assessment. The severity of injury should be recorded by medical workers or a standard instrument should be used. Lastly, since the participants were all from elementary and junior schools in 3 rural regions of Hei Longjiang Province, the findings might not be generalizable to other areas of China. Meanwhile, the data were come from an existing cohort database, the sample effect size has not been calculated.

## Conclusion

Fall, traffic injury, animal bite injury and burn are common unintentional injuries in northeast rural of China. The risk of unintentional injury increases with the number of protective factors index, and with the increase of protective factors, the effect of risk factors will be weakened. The findings of this study will contribute to the understanding of cumulative risk factors and cumulative protective factors, as well as their interaction were associated with the occurrence and/ or severity of unintentional injury in children. Pediatric care providers should target intervention in strengthening the protective factors to prevent childhood injury. Safety education in supervision, knowledge of preventing injury, retrofitting hazardous environment, and the first aid skills training should be adapted in the school through children. Pediatric care providers should target intervention in strengthening the protective factors to prevent childhood injury. Safety education in supervision, knowledge of preventing injury, retrofitting hazardous environment, and the first aid skills training should be adapted in the school through children.

## Supplementary Information

**Additional file 1.** English version of Knowledge, attitude and skills questionnaire for children unintentional injury (KAP).

**Additional file 2.** The datasets of Unintentional injury and their controls, children 6 to 14 years old, Hei Longjiang, China, 2017–2018.

## Data Availability

The datasets used and/or analyzed during this study are available from the corresponding author on reasonable request. The datasets represented in the Additional file 2.

## Abbreviations

RFI: Risk factor index
PFI: Protection factor index
RFI⨯ PFI: Interactive effect of RFI and PFI
OR: Odds ratios
CI: Confidence interval
PCUI: Protective for Childhood unintentional injury
PSAPQ: Parent Supervision Attributes Profile Questionnaire
SDQ: Strengths and Difficulties Questionnaire
IBC: Injury Behavior Checklist
HOME: Home Observation for Measurement of the Environment
KAP: Knowledge, attitude and skills for children unintentional injury
RFI (1): One risk factors
RFI (2): Two risk factors
RFI(3): Three risk factors
PFI(1): One protective factors
PFI(2): Two protective factors
