# Supplementary material for: Cumulative effect of risk and protective factors on unintentional injury for Chinese rural children: a nested case-control study
Source: BMC Public Health. 2021 Sep 23;21:1730. doi: 10.1186/s12889-021-11769-7 (PMC8461983; doi:10.1186/s12889-021-11769-7)
Supplement: Supplementary file 1 — Additional file 1. English version of Knowledge, attitude and skills questionnaire for children unintentional injury (KAP). [file 12889_2021_11769_MOESM1_ESM.pdf]

Knowledge, attitude and skills questionnaire for children unintentional injury (KAP)

Please fill in the form according to your situation in the recent year, and tick "√" under the option.	**Yes**	**None**	**Don't know**
1. Unintentional injuries are the most common cause of disability and death among children			
2. Fall injury is the most common unintentional injury in children			
3. Home is the most common place for children to suffer from unintentional injuries			
4. Do you know the common types of unintentional injuries in children (i.e. traffic injuries, falls, burns)?			
5. Children can sit in wherever in the car (i.e. motor vehicle, motorcycle, tricycle, agricultural vehicle)			
6. There should be no furniture or objects that children can climb on at home			
7. Children need to wear safe protective devices (i.e. knee pads, wrist pads, helmets, etc.) when riding scooter, roller skating or bicycle.			
8. After a fall down or sprain, movement should be reduced and cold compress for the swollen area should be applied			
9. When children bathing, it should be put cold water first, and then put hot water			
10. Children should be rinsed immediately with cold water after scalding			
11. Children should be treated after being cut or stabbed by a sharp object			
12. Pull the children out immediately after crush injury			
13. Children who are bitten by animals (cats, dogs), poultry or pets need to be vaccinated			
14. Detergent, washing powder, gasoline, pesticides and other chemical liquids should not be placed in the drinking bottles at home			
15. If a child is stuck in the throat with food or toys, first aid should be taken immediate			
16. Wires or power plugs should not be exposed at home			
17. When children drowning, help calling and first aid should be taken immediately			
18.Children should less go out when the weather is bad (i.e. rain, thunder, dust, cold)			

	**Extremely Disagree**	**Disagree**	**Neutrality**	**Agree**	**Absolutely Agree**
19. Childhood unintentional injuries can be prevented					
20.Parental care, companionship and education are very helpful in					

preventing unintentional injuries					
21. I often worry about unintentional injuries to my children					
22. It's a bad luck if my child had unintentional injuries					
23. Knowledge of the preventive of childhood unintentional injury could help to reduce the occurrence of unintentional injuries					
24. I lack first aid knowledge and skills for childhood unintentional injuries					
25. I have confidence that my child will not suffer from unintentional injury					
26.I can do nothing when my child been hurt					
27. I felt so guilty after my child's injury					
28. It is necessary to receive training in knowledge and skills of preventive unintentional injury for children					
29.I would like to participate in preventive knowledge and first aid training for the children's unintentional injury					
30. I am willing to talk to teachers and medical staff about unintentional injuries					
	**Never**	**Rare ly**	**Some times**	**Oft en**	**Alw ays**
31. You have provided safety education for children to prevent unintentional injuries (i.e. calling for help, staying away from danger)					
32. You have a first aid kit or supplies at home					
33. You have picked up and accompany with your children to go to school or go outside					
34. You will protect your child when he/she jumps from high places					
35. You will keep the floor dry and safe, and there will be no obstacles in the house which may interfere with children's activities					
36. You will prepare safety equipment for your child (i.e. knee pads, wrist pads, helmets)					
37. You will take protective measures before your child touches a hot container					
38. You will test the temperature of water before your child drinks hot water or takes a bath					
39. You don't let your children play with fire and other inflammable and explosive things					
40. You will keep sharp objects out of your child reach or in a safe place at home					
41. After your child falls or crush injury, you will do simple treatment					
42. You will take your child to get vaccinated after being bitten by animals or pets					
43. If your child takes toxic substances (i.e. detergent, washing powder, gasoline, pesticides, medicines), you will immediately send he/she to the hospital					

44. You will give home first aid to your child when a foreign object in his throat					
45. You will unplug the power immediately when your child is electrocuted					
46. You will teach your child not to swim or play alone in reservoirs, rivers or lakes.					
47. If the child does not breathe or have a heartbeat, you will perform CPR					
48.When the weather is bad (rain, thunder, dust, haze, cold), you will not permit your child to go out					
49. You will regularly maintain safety facilities at home					
